# Cerebral hemodynamic response during the resuscitation period after hypoxic-ischemic insult predicts brain injury on day 5 after insult in newborn piglets

**DOI:** 10.1038/s41598-022-16625-1

**Published:** 2022-08-01

**Authors:** Yasuhiro Nakao, Shinji Nakamura, Yinmon Htun, Tsutomu Mitsuie, Kosuke Koyano, Kenichi Ohta, Yukihiko Konishi, Takanori Miki, Masaki Ueno, Takashi Kusaka

**Affiliations:** 1 Department of Pediatrics, Faculty of Medicine, Kagawa University, 1750-1 Mikicho, Kitagun, Kagawa 761-0793 Japan; 2 Maternal Perinatal Center, Faculty of Medicine, Kagawa University, 1750-1 Mikicho, Kitagun, Kagawa 761-0793 Japan; 3 Department of Anatomy and Neurobiology, Faculty of Medicine, Kagawa University, 1750-1 Mikicho, Kitagun, Kagawa 761-0793 Japan; 4 Department of Pathology and Host Defense, Faculty of Medicine, Kagawa University, 1750-1 Mikicho, Kitagun, Kagawa 761-0793 Japan

**Keywords:** Neurology, Neurological disorders

## Abstract

Perinatal hypoxic-ischemic brain injury of neonates remains a significant problem worldwide. During the resuscitation period, changes in cerebral hemoglobin oxygen saturation (ScO_2_) have been identified by near-infrared spectroscopy (NIRS). However, in asphyxiated neonates, the relationship between these changes and brain injury is not known. Three-wavelength near-infrared time-resolved spectroscopy, an advanced technology for NIRS, allows for the estimation of ScO_2_ and cerebral blood volume (CBV). Here, we studied changes in ScO_2_ and CBV during the resuscitation period after hypoxic-ischemic insult and the relationship between these changes after insult and histopathological brain injuries on day 5 after insult using an asphyxiated piglet model. Of 36 newborn piglets subjected to hypoxic-ischemic insult, 29 were analyzed. ScO_2_ and CBV were measured 0, 5, 10, 15, and 30 min after the insult. Brain tissue was histologically evaluated on day 5. ScO_2_ and CBV increased immediately after the insult, reached a peak, and then maintained a consistent value. The increase in CBV 5 to 30 min after the insult was significantly correlated with histopathological injury scores. However, there was no correlation with ScO_2_. In conclusion, an increase in CBV within 30 min after hypoxic-ischemic insult reflects the histopathological brain injury on day 5 after insult in a piglet model.

## Introduction

Perinatal brain injury in term newborn infants mainly results from hypoxic-ischemic encephalopathy (HIE), which is a significant cause of global pediatric mortality and disability, despite the introduction of therapeutic hypothermia^1–3^. During the postnatal resuscitation period, cerebral oxygenation and hemodynamics should be assessed because the changes in these parameters during HIE may be critical determinants of brain injury severity^4^. However, the neonatal resuscitation guidelines recommend routine monitoring of heart rate (HR) and oxygen saturation by pulse oximetry/electrocardiography, which assesses only systemic oxygenation and circulation, and there is no indication for assessment of cerebral hemodynamics^5–7^.

Near-infrared spectroscopy (NIRS) allows the noninvasive continuous measurement of cerebral hemoglobin (Hb), cerebral hemoglobin oxygen saturation (ScO_2_), or the tissue oxygen index at the bedside. Furthermore, three-wavelength near-infrared time-resolved spectroscopy (TRS) is an advanced NIRS mode for measuring not only ScO_2_, but also the absolute value of cerebral blood volume (CBV), calculated from total Hb. CBV measurements using TRS have been used to monitor changes in cerebral hemodynamics after birth^8–10^.

CBV measurements are also useful in the assessment of brain injury after HIE. We reported that, in HIE neonates, high CBV values at 6 h after birth indicated adverse outcomes^10^. Moreover, in an asphyxiated piglet model, we found that the CBV increment 1–6 h after hypoxic-ischemic (HI) insult is associated with a poor outcome after insult^11^. In that and another study^12^, we also reported a CBV increment within 30 min after HI insult. Moreover, the CBV increment varied according to the degree of the insult. However, these previous studies did not address the relationship between the degree of the CBV increment within 30 min and the histopathological outcome. In clinical practice, the first 30 min after birth is considered the “critical period” for neonatal resuscitation. In the guidelines on neonatal resuscitation, if all resuscitation steps are effectively completed and there is no cardiac response by 20 min, a change in direction of care should be discussed with the team and family^13^. Therefore, in clinical practice, the assessment of brain injury at 20–30 min after birth was considered important for subsequent treatment decisions. If we can show that the changes in CBV in the piglet in the first 30 min after HI insult are predictive of brain injury severity, NIRS could be used in clinical practice to measure CBV values during the resuscitation of HIE neonates to assess insult severity. Hence, evaluation of the severity of brain injury using TRS during this resuscitation period will enable the earlier initiation of brain protective treatments such as therapeutic hypothermia and other adjunct therapies and will thus help to improve the prognosis of HIE.

In this study, using an asphyxiated piglet model, we addressed whether changes in CBV within the 30 min “resuscitation period” can indicate the severity of histopathological brain injuries on day 5.

## Results

We performed HI insult in 36 piglets. The piglets were allocated to three groups based on the results of histopathological scores. Piglets with a total histopathological score of zero were classified as undamaged. Piglets that did not survive for 5 days and died en route were classified as dead. Piglets that survived for 5 days and had histopathological damage to the brain were classified as damaged. There were 4 undamaged piglets, 19 damaged piglets, and 13 dead piglets. Two of the four undamaged piglets were excluded because the first had severe anemia (due to bleeding from the umbilical cord before preparation) and the second had instability in the TRS reading. Of the 19 damaged piglets, 2 were excluded due to either a lack of histological (HIST) data or TRS data. Of the 13 dead piglets, three were excluded because the first and the second had severe anemia (due to bleeding from the umbilical cord before preparation) and the third had respiratory failure with aspiration. Of the 10 dead piglets analyzed, eight died within 1 day after the HI insult (including one that died at 4 h post-extubation) and two piglets died on day 2 after the insult. The cause of death in all piglets was severe seizure. The piglets in this study were not euthanized but rather their deaths were sudden or accidental. Therefore, when we examined them, the piglets had been dead for some time, making brain perfusion impossible. For these reasons, we analyzed the remaining 29 piglets.

### Blood biochemistry

Blood pH, partial pressure of oxygen (PaO_2_), partial pressure of carbon dioxide (PaCO_2_), and base excess were markedly lower after HI insult than at baseline, whereas blood glucose, lactate, and Hb levels were markedly increased (Table 1).

**Table 1:** Measurements of blood gas at baseline and the end of HI insult (n = 29).

	Baseline	End of HI insult	*p* value
pH	7.46 (0.06)	6.89 (0.10)	< 0.0001
PaO_2_ (mmHg)	103 (22)	15.7 (4.7)	< 0.0001
PaCO_2_ (mmHg)	42.1 (6.0)	31.7 (9.9)	< 0.0001
BE (mmol/mL)	5.35 (2.4)	− 25.5 (2.7)	< 0.0001
Glucose (mg/dL)	153 (33)	225 (90)	< 0.0001
Lactate (mg/dL)	19.4 (15)	211 (26)	< 0.0001
Hb (g/dL)	10.2 (1.9)	10.7 (2.0)	< 0.0001

Data are presented as mean (standard deviation). Blood samples were taken about 2 h before HI insult and at the end of the insult. There was one missing data point for PaO_2_. HI, hypoxic-ischemic; pH, arterial pH; PaO_2_, arterial partial pressure of oxygen; PaCO_2_, arterial partial pressure of carbon dioxide; BE, base excess; Hb, hemoglobin.

### Physiological status of the piglets

**Figure 1:**
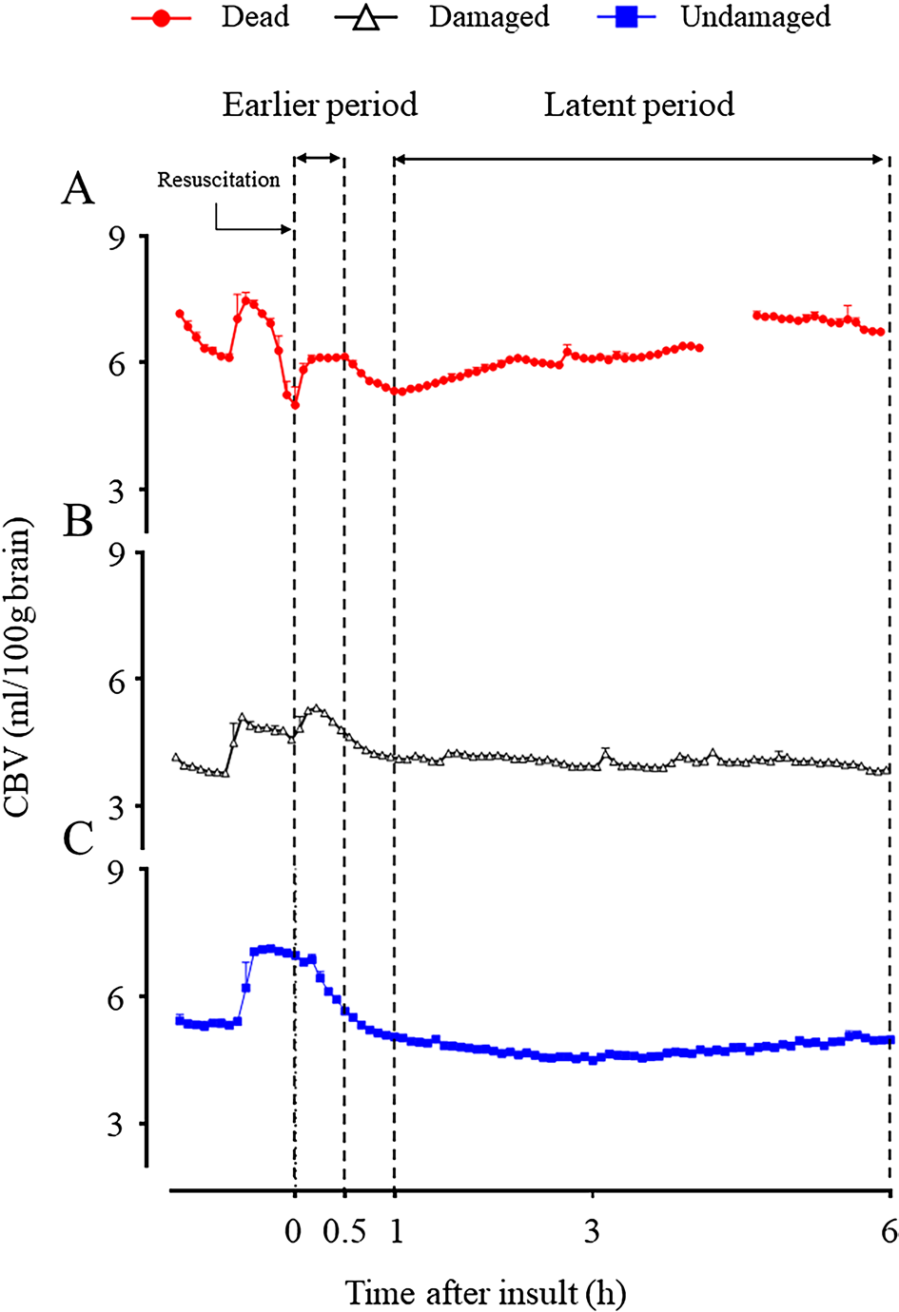
Typical pattern of changes in CBV before and after HI insult in dead, damaged, and undamaged piglets. (**A**) Typical pattern of changes in CBV in a dead piglet. CBV increased and maintained its value until 30 min after the insult. It then gradually decreased but increased again from around 1 h after the insult. (**B**) Typical pattern of changes in CBV in a damaged piglet. CBV increased once after the insult and then decreased before 30 min and did not increase again. (**C**) Typical pattern of changes in CBV in an undamaged piglet. CBV increased slightly after the insult but quickly decreased. *HI* hypoxic-ischemic, *CBV* cerebral blood volume.

Figure 1 shows representative examples of the changes in CBV before and after the HI insult in dead, damaged, and undamaged piglets. The changes in CBV after HI insult reflected the outcome. In the dead piglet group, CBV increased and maintained its value until 30 min after the insult. It then gradually decreased but increased again from around 1 h after the insult (Fig. 1A). In the damaged piglet group, CBV increased once after the insult and then decreased before 30 min and did not increase again (Fig. 1B). In the undamaged piglet group, CBV increased slightly after the insult but quickly decreased (Fig. 1C).

**Figure 2:**
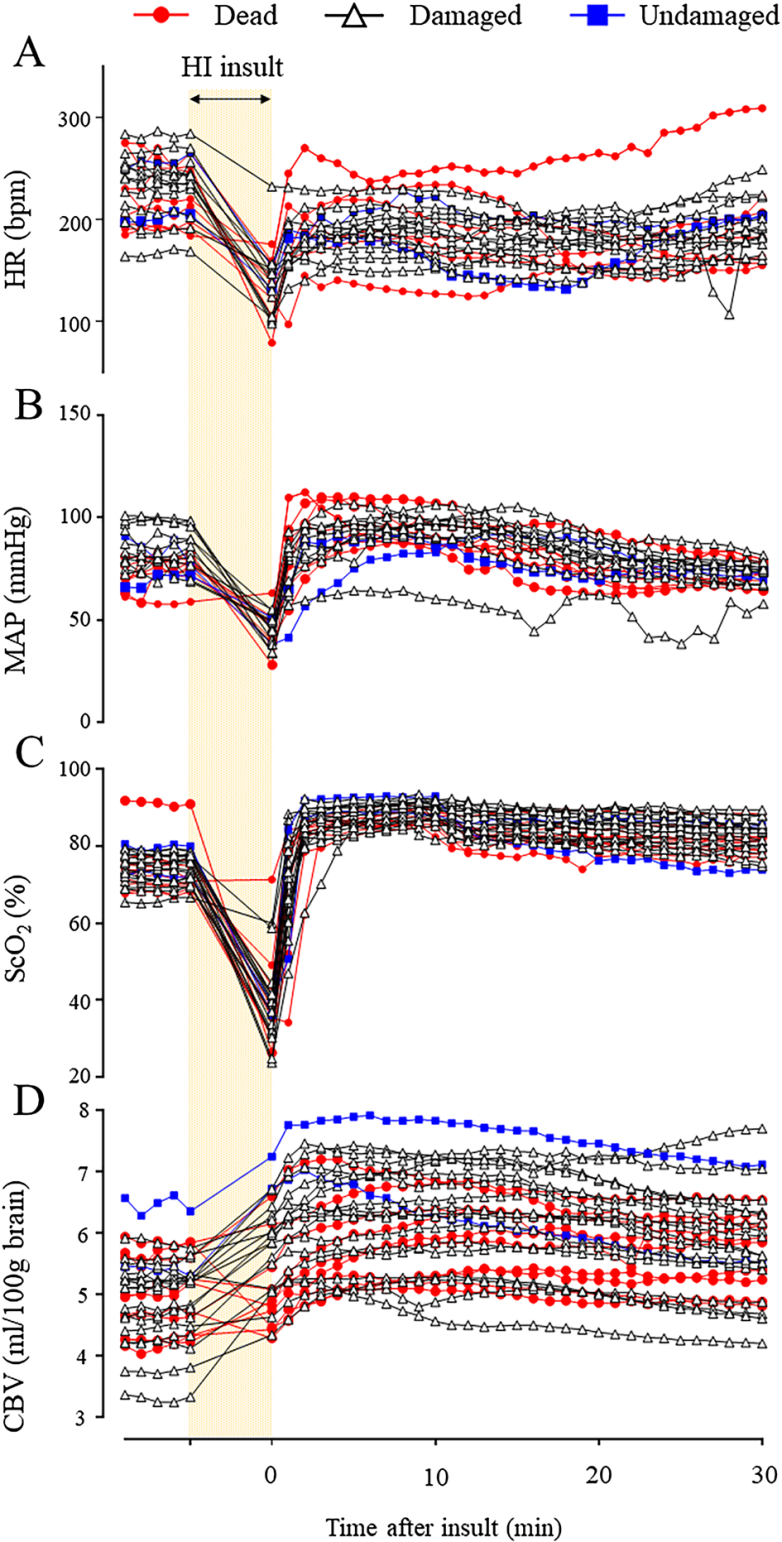
Time course of HR (**A**), MAP (**B**), ScO_2_ (**C**), and CBV(**D**) in the first 30 min after HI insult. *CBV* cerebral blood volume, *HI* hypoxic-ischemic, *HR* heart rate, *MAP* mean arterial pressure, *ScO*_*2*_ cerebral hemoglobin oxygen saturation.

Figure 2 shows the time course of the HR, mean arterial pressure (MAP), CBV, and ScO_2_ changes in the first 30 min after HI insult in all piglets. After initial resuscitation, HR values immediately increased and peaked within a few minutes or stayed the same, followed by an increase about 20 min after insult (Fig. 2A). MAP also increased immediately and peaked within 10 min, followed by a gradual decrease (Fig. 2B). For cerebral hemodynamic changes, ScO_2_ and CBV immediately increased after HI insult, before peaking and maintaining consistently high values (Fig. 2C,D).

### Piglet characteristics and histological data and scoring

Table 2 shows the physiological characteristics, outcomes, and HIST scores of each piglet. Two piglets had no histological damage, 10 piglets died, and the remaining 17 piglets survived and had histological damage. All of the dead piglets had seizures but not all of those who had seizures died. No seizures were observed in the undamaged piglets. In the 17 damaged piglets, there was no significant relationship between the presence or absence of seizures and the HIST score in any part of the brain.

**Table 2:** Piglet characteristics and histological data and scoring (n = 29).

	Sex	BW (g)	Presence of clinical seizures	Outcome	WM	GM	HIPP	CERE
1	M	1400	−	Undamaged	0.0	0.0	0.0	0.0
2	M	1760	−	Undamaged	0.0	0.0	0.0	0.0
3	F	2100	+	Damaged	3.8	3.8	3.2	0.0
4	F	1720	−	Damaged	2.2	1.9	1.2	0.4
5	M	1900	−	Damaged	3.6	3.4	3.8	0.0
6	F	1800	+	Damaged	3.8	3.8	4.0	2.8
7	F	1800	+	Damaged	2.6	2.0	1.0	0.0
8	M	1630	−	Damaged	3.6	3.6	3.4	0.6
9	M	2000	−	Damaged	3.6	3.6	4.0	2.0
10	M	1640	−	Damaged	3.0	3.3	3.8	1.8
11	M	1670	+	Damaged	4.0	4.0	4.0	2.0
12	M	1900	−	Damaged	3.0	1.0	2.0	0.0
13	M	1910	+	Damaged	4.0	3.0	4.0	1.0
14	M	1800	+	Damaged	0.0	1.0	0.0	0.0
15	F	1780	−	Damaged	3.8	4.0	4.0	0.0
16	F	1950	−	Damaged	1.0	3.0	3.0	0.0
17	F	2000	+	Damaged	3.0	4.0	3.0	3.0
18	M	1700	−	Damaged	1.0	3.0	1.0	1.0
19	F	1760	+	Damaged	1.3	3.4	1.2	1.2
20	M	2000	+	Dead	4.0	4.0	4.0	4.0
21	F	1850	+	Dead	4.0	4.0	4.0	4.0
22	M	1600	+	Dead	4.0	4.0	4.0	4.0
23	F	1810	+	Dead	4.0	4.0	4.0	4.0
24	M	1790	+	Dead	4.0	4.0	4.0	4.0
25	F	1710	+	Dead	4.0	4.0	4.0	4.0
26	F	1870	+	Dead	4.0	4.0	4.0	4.0
27	M	1700	+	Dead	4.0	4.0	4.0	4.0
28	M	1810	+	Dead	4.0	4.0	4.0	4.0
29	M	1750	+	Dead	4.0	4.0	4.0	4.0

M, male; F, female; BW, body weight; WM, white matter; GM, gray matter; HIPP, hippocampus; CERE, cerebellum.

### Changes in CBV after HI insult

**Figure 3:**
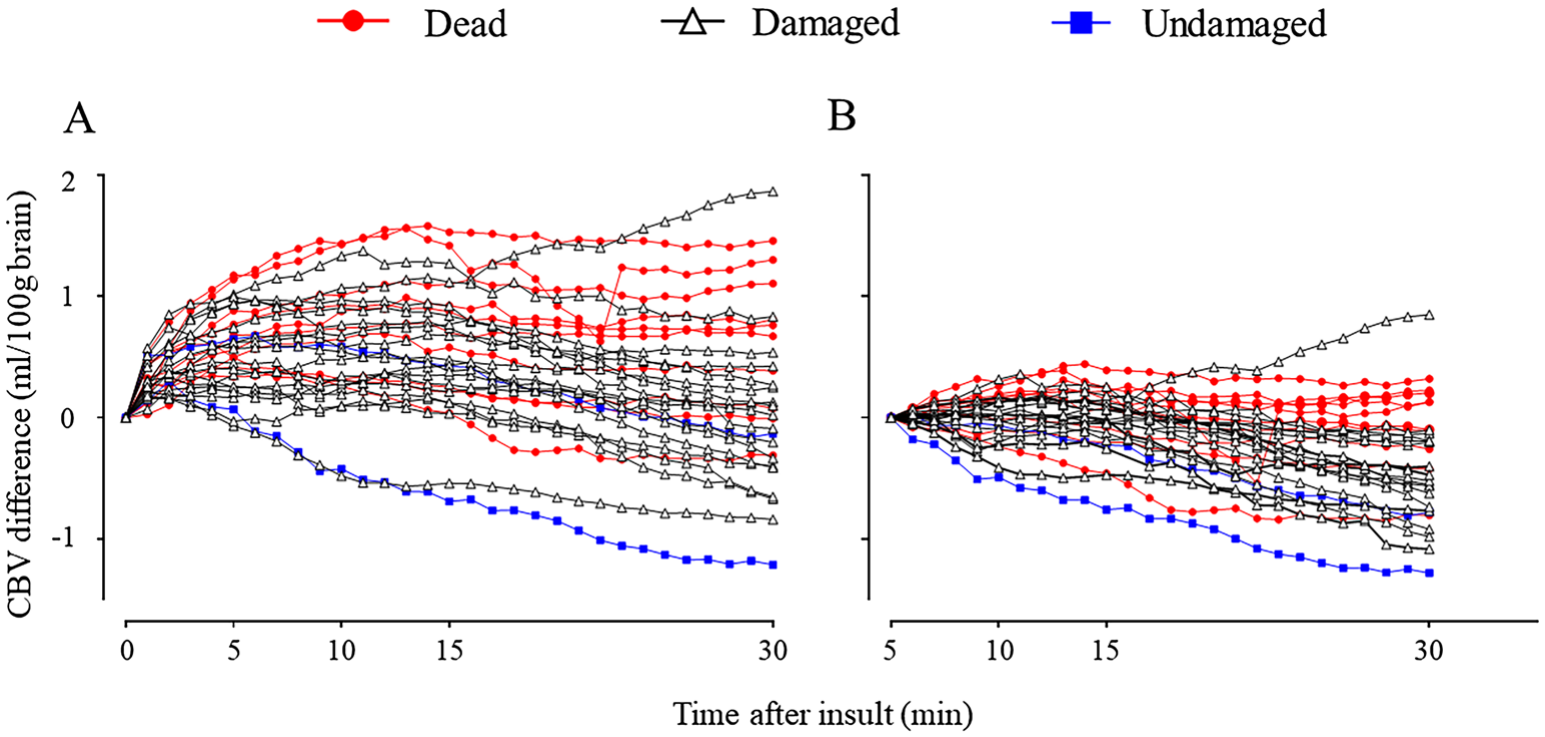
The difference in CBV from after the HI insult to each time point (**A**) and from 5 min after the insult to each time point (**B**). Both immediately after HI insult and 5 min after insult, most of the temporal changes in CBV in each piglet overlapped, regardless of the group. *CBV* cerebral blood volume, *HI* hypoxic-ischemic.

Absolute CBV varied substantially among individual piglets (Fig. 2D). As in the typical case shown in Fig. 1, we considered that the pattern of the changes in CBV within 30 min reflected the outcome. However, we concluded that outcome could not be determined based on the CBV pattern alone because most of the temporal changes in CBV in each piglet overlapped, regardless of the group (Fig. 3). Thus, we examined whether the degree of the change in CBV, rather than the pattern, reflected the outcome: we calculated the differences in CBV from the end of the insult and 5 min after the HI insult to each time point until 30 min and investigated the relationship between these differences in CBV and the histological brain injury using the HIST score.

### Relationship of the changes in HR, MAP, ScO_2_, and CBV with HIST score

Table 3 shows the relationship of the changes in CBV and ScO_2_ 5, 10, 15, and 30 min after HI insult with the HIST scores of the respective brain regions (white matter [WM], gray matter [GM], hippocampus [HIPP], and cerebellum [CERE]) on day 5 after insult. At 10 and 15 min, CBV was significantly correlated with HIST scores in the WM, GM, and HIPP. Furthermore, at 30 min, there was a significant correlation of CBV with HIST scores in the WM, GM, HIPP, and CERE. Table 4 shows the relationship between the changes in CBV, ScO_2_, HR, and MAP 10, 15, and 30 min from 5 min after HI insult and the HIST scores of the respective brain regions on day 5 after insult. At 5 to 10, 5 to 15 min, and 5 to 30 min, CBV was significantly correlated with HIST scores in the WM, GM, HIPP, and CERE. In other words, a larger increase in CBV indicated more severe brain injury. As shown in the tables, these correlations tended to become stronger over time. In addition, in piglets with an increased CBV, CBV remained higher in those with worse histopathological damage and showed a tendency for a decrease in those with less damage. The same analysis was performed for changes in HR, MAP, and ScO_2_. For MAP, there was a significant correlation with the HIST scores in the WM at 5 to 10 min and 5 to 30 min and in the CERE at 5 to 10 min and 5 to 15 min; for ScO_2_, the HIST scores in the HIPP at 5 to 30 min exhibited a significant correlation (Tables 3 and 4). In addition, the same analysis was performed excluding dead piglets (n = 19) (Tables 5 and 6). These results showed that the correlation between CBV and HIST scores was similar to that in Tables 3 and 4. However, for ScO_2_, a correlation was observed for the changes in ScO_2_ from 5 to 30 min, except in the GM. In the dead piglet group (n = 10), there was no correlation of the CBV, ScO_2_, HR, and MAP with HIST scores.

**Table 3:** Correlation coefficients between changes in CBV and ScO_2_ from the endpoint of HI insult and HIST scores on day 5 in different brain regions in all piglets (n = 29).

	0–5 min		0–10 min		0–15 min		0–30 min	
	r	*p*	r	*p*	r	*p*	r	*p*
**CBV**								
WM	0.525	0.0034**	0.616	0.0004***	0.562	0.0015**	0.713	< 0.0001****
GM	0.367	0.0503	0.469	0.0103*	0.396	0.0335*	0.624	0.0003***
HIPP	0.338	0.0729	0.448	0.0147*	0.381	0.0417*	0.573	0.0012**
CERE	0.294	0.1212	0.380	0.0419*	0.328	0.0826	0.502	0.0055**
**ScO**_**2**_								
GM	− 0.064	0.7402	− 0.101	0.6029	− 0.089	0.6456	− 0.000	0.9989
WM	− 0.191	0.3198	− 0.254	0.1843	− 0.284	0.1349	− 0.170	0.3774
HIPP	− 0.093	0.6296	− 0.139	0.4733	− 0.146	0.4497	− 0.013	0.9477
CERE	− 0.019	0.9235	− 0.094	0.6276	− 0.124	0.5210	− 0.026	0.8922

0–5 min, 0–10 min, 0–15 min, and 0–30 min indicate changes in CBV and ScO_2_ from the endpoint of HI insult to 5 min, 10 min, 15 min, and 30 min, respectively.

CBV, cerebral blood volume; ScO_2_, cerebral hemoglobin oxygen saturation; HI, hypoxic-ischemic; HIST, histological; WM, white matter; GM, gray matter; HIPP, hippocampus; CERE, cerebellum.

*p < 0.05; **p < 0.01; ***p < 0.001; ****p < 0.0001; Spearman’s rank correlation coefficient was used for nonparametric data.

**Table 4:** Correlation coefficients between changes in CBV, ScO_2_, HR, and MAP from 5 min after the endpoint of HI insult and HIST scores on day 5 in different brain regions in all piglets (n = 29).

	5–10 min		5–15 min		5–30 min	
	r	*p*	r	*p*	r	*p*
**CBV**						
WM	0.524	0.0036**	0.519	0.0039**	0.686	< 0.0001****
GM	0.458	0.0126*	0.409	0.0275*	0.645	0.0002***
HIPP	0.416	0.0247*	0.377	0.0441*	0.580	0.0009***
CERE	0.428	0.0207*	0.407	0.0284*	0.569	0.0013**
**ScO**_**2**_						
WM	0.042	0.8274	0.193	0.3150	0.352	0.0613
GM	− 0.037	0.8474	0.100	0.6074	0.277	0.1462
HIPP	0.038	0.8439	0.188	0.3294	0.439	0.0173*
CERE	− 0.120	0.5341	0.047	0.8107	0.263	0.1674
**HR**						
WM	− 0.124	0.5824	− 0.066	0.7696	0.070	0.7569
GM	− 0.041	0.8571	0.116	0.6069	0.236	0.2901
HIPP	− 0.055	0.8089	0.057	0.8026	0.169	0.4515
CERE	− 0.099	0.6609	− 0.056	0.8035	0.064	0.7775
**MAP**						
WM	− 0.433	0.0443*	− 0.333	0.1304	− 0.522	0.0126*
GM	− 0.224	0.3169	− 0.128	0.5711	− 0.335	0.1270
HIPP	− 0.232	0.2998	− 0.135	0.5503	− 0.400	0.0650
CERE	− 0.608	0.0027**	− 0.490	0.0208*	− 0.345	0.1156

5–10 min, 5–15 min, and 5–30 min indicate changes in CBV, ScO_2_, HR, and MAP from 5 min after the endpoint of HI insult to 10 min, 15 min, and 30 min, respectively. For HR and MAP, six piglets were missing data.

CBV, cerebral blood volume; ScO_2_, cerebral hemoglobin oxygen saturation; HR, heart rate; MAP, mean arterial pressure; HI, hypoxic-ischemic; HIST, histological; WM, white matter; GM, gray matter; HIPP, hippocampus; CERE, cerebellum.

*p < 0.05; **p < 0.01; ***p < 0.001; ****p < 0.0001; Spearman’s rank correlation coefficient was used for nonparametric data.

**Table 5:** Correlation coefficients between changes in CBV and ScO_2_ from the endpoint of HI insult and HIST scores on day 5 in different brain regions after the exclusion of dead piglets (n = 19).

	0–5 min		0–10 min		0–15 min		0–30 min	
	r	*p*	r	*p*	r	*p*	r	*p*
**CBV**								
WM	0.524	0.0213*	0.652	0.0025**	0.681	0.0013**	0.686	0.0012**
GM	0.340	0.1544	0.488	0.0339*	0.434	0.0637	0.624	0.0043**
HIPP	0.288	0.2324	0.419	0.0741	0.384	0.1042	0.533	0.0189*
CERE	0.156	0.5238	0.217	0.3717	0.225	0.3552	0.253	0.2970
**ScO**_**2**_								
WM	− 0.125	0.6095	− 0.127	0.6044	0.034	0.8917	0.032	0.8973
GM	− 0.333	0.1637	− 0.372	0.1171	− 0.228	0.3482	− 0.171	0.4832
HIPP	− 0.118	0.6302	− 0.116	0.6354	− 0.012	0.9597	0.063	0.7977
CERE	− 0.026	0.9159	− 0.090	0.7139	− 0.010	0.9669	0.030	0.9039

0–5 min, 0–10 min, 0–15 min, and 0–30 min indicate changes in CBV and ScO_2_ from the endpoint of HI insult to 5 min, 10 min, 15 min, and 30 min, respectively.

CBV, cerebral blood volume; ScO_2_, cerebral hemoglobin oxygen saturation; HI, hypoxic-ischemic; HIST, histological; WM, white matter; GM, gray matter; HIPP, hippocampus; CERE, cerebellum.

*p < 0.05; **p < 0.01; Spearman’s rank correlation coefficient was used for nonparametric data.

**Table 6:** Correlation coefficients between changes in CBV, ScO_2_, HR, and MAP from 5 min after the endpoint of HI insult and HIST scores on day 5 in different brain regions after the exclusion of dead piglets (n = 19).

	5–10 min		5–15 min		5–30 min	
	r	*p*	r	*p*	r	*p*
**CBV**						
WM	0.528	0.0201*	0.619	0.0047**	0.624	0.0043**
GM	0.487	0.0343*	0.455	0.0504	0.627	0.0041**
HIPP	0.382	0.1068	0.368	0.1216	0.519	0.0230*
CERE	0.279	0.2467	0.370	0.1195	0.316	0.1881
**ScO**_**2**_						
WM	0.188	0.4413	0.434	0.0635	0.467	0.0441*
GM	0.082	0.7382	0.312	0.1939	0.403	0.0874
HIPP	0.206	0.3976	0.426	0.0689	0.616	0.0050**
CERE	− 0.103	0.6747	0.252	0.2988	0.465	0.0448*
**HR**						
WM	− 0.171	0.5546	− 0.068	0.8164	− 0.217	0.4508
GM	− 0.056	0.8474	0.205	0.4783	0.195	0.5008
HIPP	− 0.055	0.8514	0.127	0.6624	0.141	0.6274
CERE	− 0.165	0.5682	− 0.054	0.8547	− 0.239	0.4048
**MAP**						
WM	− 0.185	0.5231	− 0.227	0.4298	− 0.662	0.0119*
GM	0.287	0.3172	0.245	0.3936	− 0.373	0.1883
HIPP	0.170	0.5590	0.118	0.6835	− 0.401	0.1548
CERE	− 0.555	0.0423*	− 0.502	0.0697	− 0.268	0.3498

5–10 min, 5–15 min, and 5–30 min indicate changes in CBV, ScO_2_, HR, and MAP from 5 min after the endpoint of HI insult to 10 min, 15 min, and 30 min, respectively. For HR and MAP, four piglets were missing data.

CBV, cerebral blood volume; ScO_2_, cerebral hemoglobin oxygen saturation; HR, heart rate; MAP, mean arterial pressure; HI, hypoxic-ischemic; HIST, histological; WM, white matter; GM, gray matter; HIPP, hippocampus; CERE, cerebellum.

*p < 0.05; **p < 0.01; Spearman’s rank correlation coefficient was used for nonparametric data.

## Discussion

Here, we examined the changes in CBV and ScO_2_ in the first 30 min, considered the critical resuscitation period, after HI insult and the relationship between these changes and the histological brain injury on day 5 after insult. We found that the changes in CBV from the endpoint of the HI insult and 5 min after the insult to 30 min after the insult were correlated with the histopathological brain injury on day 5 after insult: the greater the increase in CBV, the more severe the histological brain injury.

An increase in CBV in the first 30 min after HI insult, considered the earlier period and reflective of transient cerebral hyperemia, may be caused by vasodilatation due to the accumulation of lactic acid and other vasoactive substances such as ADP and AMP during ischemia^14–16^. We previously reported that the CBV increase 6 and 12 h after HI insult reflected severe brain injury in newborn piglets^17^, as in this study, and Marks et al. reported that a larger increase in cerebral total hemoglobin (totalHb) within 6 h after HI insult was associated with more severe cerebral injury^18^. These results indicate that the CBV increase after HI insult reflects higher cerebral blood flow (CBF) due to impaired cerebral autoregulation^4^ and that piglets with impaired cerebral autoregulation cannot maintain adequate CBF to meet oxygen demand in the brain, culminating in permanent brain injury.

The CBV responses identified here may reflect the cerebral hemodynamic conditions during HI insult. HI insults can be divided into an “initial compensation period” and a subsequent progressive “HI decompensation period” and culminate in profound systemic hypotension with cerebral hypoperfusion^19,20^. When the initial compensation period is before the initial resuscitation, CBF can be maintained without severe brain hypoperfusion during the insult and cerebral autoregulation remains intact. In this case, although the systemic blood pressure and HR rise immediately after the initial resuscitation, the CBF remains stable owing to unimpaired cerebral autoregulation, leading to less cerebral reperfusion and a lower CBV increase. However, when the HI decompensation period is before the initial resuscitation, cerebral autoregulation can be severely impaired. After the initial resuscitation, CBF passively rises due to an immediate increase in systemic blood pressure and HR. We reported, using the asphyxiated piglet model, that the decrease in CBV during HI insult was correlated with the increase in CBV at 1 h after insult^12^. In addition, fetal lamb studies linked the hypotensive period during severe asphyxia with cerebral injury across paradigms, likely because of the close relationship between the maintenance of fetal blood pressure during severe asphyxia and changes in brain perfusion^19,20^.

There have been many reports on ScO_2_ changes in the earlier period^21–24^, suggesting the value of cerebral oxygenation monitoring for evaluating the oxygen delivery/demand balance. In this animal study, we used TRS to measure both CBV and ScO_2_. However, the changes in ScO_2_ did not reflect brain injury, as also found for HR and MAP. It is unclear why ScO_2_ showed a consistently high value after a transient increase in the first 30 min after HI insult, whereas CBV showed various changes. ScO_2_ was calculated as the oxyhaemoglobin (oxyHb):totalHb ratio. We speculate that, because arterial vasodilation might more strongly increase not only totalHb, but also oxyHb in the earlier period with both impaired and intact cerebral autoregulation, its ratio and the ScO_2_ increase might not differ among all piglets.

Although the Neonatal Cardio-Pulmonary Resuscitation (NCPR) guidelines recommend the monitoring of HR and arterial oxygen saturation (SpO_2_) after birth^7,13,25,26^, these post-birth changes may not always be useful for predicting brain injury severity in the clinical setting. NIRS has the potential to enable easy, non-invasive monitoring of cerebral hemodynamic changes and assessment of the severity of brain injuries immediately after birth compared with other metrics such as HR and SpO_2_. Furthermore, another important feature of NIRS is that, unlike the case with other instruments such as magnetic resonance imaging (MRI) machines, the patient does not need to be moved to a different room to use NIRS, which means that resuscitation can continue in the delivery room while measurements are being taken, thereby allowing assessment of brain injury while resuscitation is performed. Although MRI and electroencephalography (EEG) are useful for predicting brain injury, they are difficult to perform during resuscitation. In mature piglets, global hemodynamic parameters are poorly correlated with tissue oxygenation/perfusion parameters during asphyxial cardiac arrest and resuscitation^27^. Hence, cerebral hemodynamic parameters such as CBV cannot be replaced with other global hemodynamic parameters. The monitoring of CBV changes from immediately after birth in neonates with asphyxia should help to differentiate brain damage severity. NIRS helps to explain the cerebral hemodynamics in neonates in the postnatal transition period^4^. In the future, CBV monitoring during the transition period might clarify postnatal brain injury severity and boost initial neuroprotective therapies such as therapeutic hypothermia.

In the clinical setting, it is difficult to measure CBV immediately after birth and there is a time lag until CBV measurement can be started. This study showed that a CBV increase from 5 min after insult can detect brain injury. Monitoring of cerebral oxygenation and hemodynamics in the 30 min after birth, in the earlier period, is critical for evaluating the balance of oxygen delivery and demand in the brain. Morimoto et al. and Schwaberger et al. reported a decreased CBV in the first 15 min after birth in healthy vaginally delivered neonates^8^ and in those delivered by cesarean section^28^. Furthermore, Morimoto et al. reported a greater CBV decrement in vaginal deliveries than in cesarean sections during the first 15 min after birth in healthy neonates^9^. Primary damage sustained in the earlier period after birth may be estimated by evaluating changes in the cerebral hemodynamic and oxygen metabolism early after resuscitation. Hence, an initial diagnosis and treatment for HIE during the critical resuscitation period can improve the prognosis for HIE neonates.

There are several limitations to this work. First, our experimental population was small. Furthermore, we investigated CBV and ScO_2_, which can be measured simply and sequentially, but could not examine direct parameters related to the cerebral circulation or oxygen metabolism, such as CBF or cerebral metabolism ratio of oxygen (CMRO_2_). We also histopathologically evaluated the brain with hematoxylin and eosin staining alone. An additional staining technique should be used to more accurately investigate the brain damage. Changes in CBV after HI insult can be used to determine the severity of brain injury. In clinical practice, using TRS, the monitoring of CBV immediately after birth may help to evaluate the severity of the brain injury in neonates with HIE.

## Methods

### Ethical approval and informed consent

This study was conducted with the approval of the Kagawa University Animal Care and Use Committee (15070-1) and in accordance with Animal Research: Reporting In Vivo Experiments (ARRIVE) guidelines. The study was carried out in compliance with the ARRIVE guidelines. All methods were carried out in accordance with relevant guidelines and regulations.

### Animal preparation

Thirty-six newborn piglets (22 males, 14 females; body weight, 1400–2100 g) were anaesthetized and prepared for surgery. They were born within 24 h after the mother went into labor and at term gestation (term piglets have a gestational age of 16 weeks). Prior to the start of the experimental procedures, the piglets were placed under a radiant warmer and briefly observed for activity and alertness. Anesthesia was induced via inhalation of 1–2% isoflurane (Forane^®^ inhalant liquid; Abbott Co., Tokyo, Japan) with the use of a facemask. Each piglet was intubated and mechanically ventilated using an infant ventilator. The umbilical vein and artery were cannulated with a 3- or 4-Fr neonatal umbilical catheter (Atom Indwelling Feeding Tube for Infants; Atom Medical Co., Tokyo, Japan). The umbilical vein catheter was placed at a site 5 cm deep from the incision and the umbilical artery catheter was placed at a site 10 cm deep from the incision for blood pressure monitoring and blood sampling, respectively. After cannulation, the piglets were anesthetized with fentanyl citrate at an initial dose of 10 μg/kg followed by continuous infusion at 5 μg/kg/h and were paralyzed with pancuronium bromide at an initial dose of 100 μg/kg followed by continuous infusion at 100 μg/kg/h. Maintenance solution (electrolytes plus 2.7% glucose [KN3B]; Otsuka Pharmaceutical Co., Tokyo, Japan) was continuously infused at 4 mL/kg/h via the umbilical vein (glucose was infused at 2 mg/kg/min). Arterial blood was sampled at critical points and when clinically indicated during the experiment. Each piglet was then placed in a copper mesh-shielded cage under a radiant warmer to maintain a rectal temperature of 38.0 ± 0.5 °C. Inspired gas was prepared by mixing O_2_ and N_2_ gases to obtain the required oxygen concentrations. Ventilation was adjusted to maintain PaO_2_ and PaCO_2_ within their normal ranges. Arterial blood pressures were measured and recorded via the umbilical arterial catheter.

### Time-resolved near-infrared spectroscopy and analysis

The probes of a portable three-wavelength TRS system (TRS-10; Hamamatsu Photonics K.K., Hamamatsu, Japan) were attached to the head of each piglet. The light emitter and detector optodes were positioned on the parietal region of each piglet with a 30-mm interoptode distance. In the TRS system, a time-correlated single-photon-counting technique is used for detection. The concentrations of oxyHb and deoxyhaemoglobin (deoxyHb) were calculated from the absorption coefficients of oxyHb and deoxyHb, with background absorption assumed to be due only to 85% (by volume) water. The total cerebral Hb concentration, ScO_2_, and CBV were calculated as described previously^29–31^.

### Amplitude-integrated electroencephalography

Neural activity was measured by amplitude-integrated electroencephalography (aEEG) (Nicolet One; Cardinal Health, Inc., Dublin, OH). All electrical devices and the copper mesh shield were grounded. The signal was displayed on a semi-logarithmic scale at low speed (6 cm/h). We conducted measurements every second. Gold-plated electrode needles were placed at the P3 and P4 positions, which corresponded to the left and right parietal regions of the head. A maximum amplitude < 5 µV was defined as low-amplitude integrated electroencephalography (LAEEG).

### Hypoxic-ischemic insult protocol

Because the details were reported in our previous studies^11,31–33^, the HI insult protocol is only outlined here. Hypoxia was induced by reducing the inspired oxygen concentration of the ventilator to 3%–4% after at least 120 min of stabilization from the initial anesthetic induction. To obtain the LAEEG pattern (< 5 μV), the inspired oxygen concentration was reduced further if required; care was taken to avoid cardiopulmonary arrest. From the beginning of LAEEG, LAEEG was maintained for 20 min. Fraction of inspiratory oxygen (FiO_2_) was decreased (0.01 decrements) or increased (0.01 increments) during the HI insult to maintain LAEEG, HR (> 130 beats/min), and MAP (> 70% of baseline). After LAEEG for 20 min, hypotension was induced by decreasing FiO_2_ until MABP was < 70% of the baseline and was maintained for 10 min. Consequently, the total LAEEG duration during the insult was 30 min. Hypoxia was terminated by resuscitation with 100% oxygen. NaHCO_3_ was used to correct a base deficit (base excess below − 5.0 mEq/L) to maintain a pH of 7.3–7.5. After 10 min of 1.0 FiO_2_, the ventilator rate and FiO_2_ were gradually reduced to maintain SpO_2_ of 95%–98%.

### Posthypoxic-ischemic insult treatment

Once the piglets were weaned off the anesthesia and ventilator and extubated 7–24 h after HI insult, they were allowed to recover and survive for 5 days in an incubator. They were fed 50–100 mL of artificial animal milk via a nasogastric tube every 6 h. The temperature of the incubator was maintained at 28–32 °C. Seizure presence was recognized clinically as rhythmic pathologic movements (cycling) and tonic postures sustained between cycling episodes. If seizures occurred, the piglet was treated with intramuscular phenobarbital (20 mg/kg). If seizures persisted, the piglet was treated with two successive anticonvulsant doses. If seizures persisted after two successive anticonvulsant doses, the piglet was euthanized.

### Histological assessment

On day 5 after HI insult, each animal’s brain was perfused with 0.9% saline and 4% phosphate-buffered paraformaldehyde. The brain tissue was histologically evaluated, and irregularities were graded according to a validated histopathology grading scale for a piglet model of posthypoxic encephalopathy^11,32,34^. Coronal blocks of WM, GM, HIPP, and CERE were embedded in paraffin and cut with a microtome at 4 µm. At regular intervals, three sections of each region were examined. For hematoxylin and eosin staining, the extent of the damage in each of the four regions was graded in 0.5-unit intervals on a 9-step scale that ranged from 0.0 to 4.0 as follows: grade 0, no damage; grade 1, ≤ 10% of the area affected with morphological changes including individual necrotic neurons and small patchy, complete or incomplete infarcts; grade 2, 20–30% of the area affected with partly confluent incomplete or complete infarcts; grade 3, 40–60% of the area affected with large confluent complete infarct; and grade 4, > 75% of the area affected with neuronal necrosis in the hippocampus and total disintegration of the cortex^11,34^. In the calculations, we awarded the maximal score of 4.0 for each region (cortical WM, 4.0; cortical GM, 4.0; HIPP, 4.0; CERE: 4.0) to the experimental piglets that failed to survive for 5 days because of severe seizures^35^.

### Statistical analysis

We defined the period during which the CBV increased during this critical resuscitation period after insult—from the end of the HI insult to 30 min after the insult—as the “earlier period” and the period during which the CBV increased again after the first increment—from 1 to 6 h after the HI insult—as the “latent period” (Fig. 1).

The final sample size was 29. Values are expressed as mean ± standard deviation for blood gas data, whereas the median with interquartile range is used for histological scores. Spearman’s rank correlation coefficient was used for nonparametric data such as the HIST scores, and the Wilcoxon signed-rank test was used for blood gas differences between pre- and post-insult effects. A p-value < 0.05 was considered to indicate statistical significance. All statistical analyses were performed using GraphPad Prism 7.02 (GraphPad Software, La Jolla, CA).

## Data Availability

The datasets generated during and/or analyzed during the current study are available from the corresponding author on reasonable request.
